# A case report of multi-ligaments injury of the ACL-MCL-PT combined with an occult fracture of the posterolateral tibial plateau

**DOI:** 10.1016/j.tcr.2021.100457

**Published:** 2021-03-17

**Authors:** Tao Xie, Xiao Han, Shao-bo Zhou, Liu-long Zhu, Qi-fang He

**Affiliations:** Department of Orthopaedic Surgery, Affiliated Hangzhou First People's Hospital of Zhejiang University School of Medicine, 261 Huansha Road, Hangzhou 310006, China

**Keywords:** Anterior cruciate ligament, Medial collateral ligament, Patellar tendon, Tibial plateau fracture

## Abstract

The anterior cruciate ligament and medial collateral ligament are important static stabilizers of the knee. The patellar tendon is part of the knee extensor mechanism. The injury simultaneously involving these three structures is very rare. This paper reports a case with simultaneous ipsilateral rupture of the anterior cruciate ligament, medial collateral ligament, patellar tendon, and an occult compression fracture of the posterolateral tibial plateau. This injury pattern has not been reported in literature yet. The injury mechanism was hypothesized as a sudden anterior translation and valgus of the proximal tibia when the knee was in high flexion, followed by an eccentric quadriceps' contracture. In the followed management, ruptured medial collateral ligament and patellar tendon were sutured with augment, while the torn anterior cruciate ligament and fracture were treated conservatively. The outcome of the treatment was satisfactory, and no complication was observed. To this combined injury, a comprehensive consideration, including physical examination, multiple imaging examinations, and analysis of injury mechanism, is essential for a full diagnosis and treatment decision. Especially, computed tomography may help to identify an occult or non-displaced fracture, which would be easily misdiagnosed when nothing unusual was found in routine X-rays. In the treatment, it is suggested to perform a selective or step-by-step repair to the damaged structures, rather than an immediate total repair after injury.

## Introduction

The knee joint is stabilized with surrounding ligaments, muscles, and tendons. Structurally, anterior cruciate ligament (ACL) and medial collateral ligament (MCL) are essential static stabilizers in knee function. Patellar tendon (PT), as a component of the knee extensor mechanism, plays a crucial role in transmitting the strength of the quadriceps muscle to the leg for walking. Isolated rupture of the ACL, MCL or PT is not uncommon, especially in contact or non-contact injury during the sports. However, a combined ACL-MCL-PT disruption is rare [1,2]. Until now, this compound injury was considered as a pure ligamentous disruption, with intact bone structure around the knee. We report a case with simultaneous ipsilateral rupture of the anterior cruciate ligament, medial collateral ligament, patellar tendon, and an occult fracture of the posterolateral tibial plateau. The content of the report consists of diagnosis, injury mechanism, and treatment.

## Case presentation

**Fig. 1:**
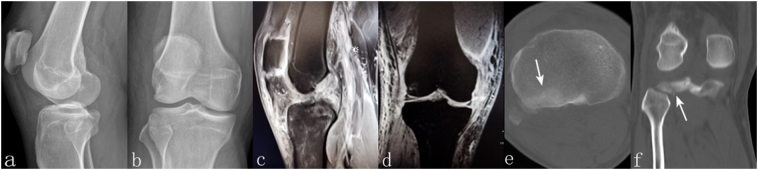
The patella alta was observed from lateral view of radiology, and there was no finding of obvious bony lesion (a, b). The ruptures of the PT, ACL, and MCL were confirmed by the MRI images (c, d). Depression and split of the posterolateral tibial plateau (white arrow) were noted on transverse and sagittal cuts of the CT scan (e, f).

The case presentation is based on the patient's consent of publication about all injury-related information. The patient, a 46-year-old male, office worker, without a medical history of patellar tendinopathy, was taken to hospital with the injury of the right knee after a scooter accident. The patient heard a “popping” sound and felt like the knee joint was “giving way” immediately. Clinical examination revealed a swollen and immobile knee. There was no open wound or symptom of neurovascular disorders. The radiography showed no obvious fracture but significant patella alta was observed (Fig. 1a, b). Magnetic Resonance Imaging (MRI) during hospitalization confirmed the simultaneous ruptures of the ACL, the MCL, and the PT (Fig. 1c, d). Both superficial and deep layers of the MCL were damaged, and the anteromedial bundle and posterolateral bundle of the ACL were torn. Lateral and medial meniscus remained intact. Undisplaced fracture of the posterolateral tibial plateau was noted by the CT(Computer Tomography) scan (1e, 1f).

**Fig. 2:**
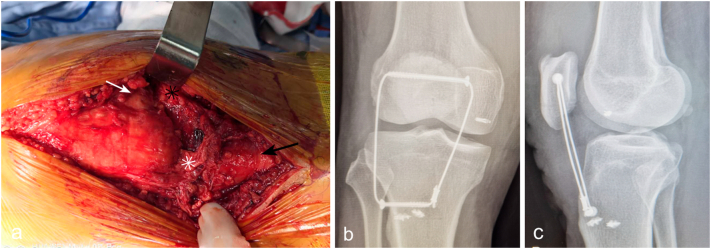
Full tear of the PT and MCL (a). The PT and MCL were repaired with suture anchor, and a protection cable was used (b, c). White arrow: medial condyle of femur; Black arrow: tibial tubercle; White star mark: patella tendon stump; Black star mark: MCL stump.

The injured limb was stabilized with the cast until definitive surgery at 1 week after the injury. Under spinal anesthesia, the physical examination showed the Lachman test (++) and valgus stress test (+++). Through a longitudinal anterior midline incision, full-thickness tears of the PT and the MCL were exposed (Fig. 2a). The torn patella tendon was reattached with Krackow suture, protected by a load-sharing cable. The MCL was repaired with suture augmentation (Fig. 2b, c). The ACL rupture and the posterolateral tibial plateau fracture were conservatively treated with a hinged ACL brace for 2 months.

The patient was instructed to perform isometric muscle training in full extension of the knee. The passive and active motion was started 7 days after surgery, permitted from 0 to 90°. The patient was not allowed to walk with full weight-bearing in the first month, preventing further compression of the fracture.

**Fig. 3:**
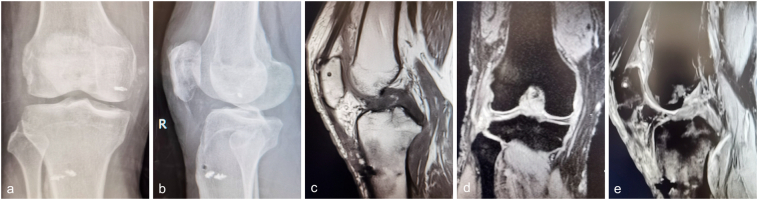
The protection cable was removed in three months after primary repair of the ligaments (a, b). The continuity of the PT and MCL was restored (c, d). The ACL healed with cicatrization (e).

**Fig. 4:**
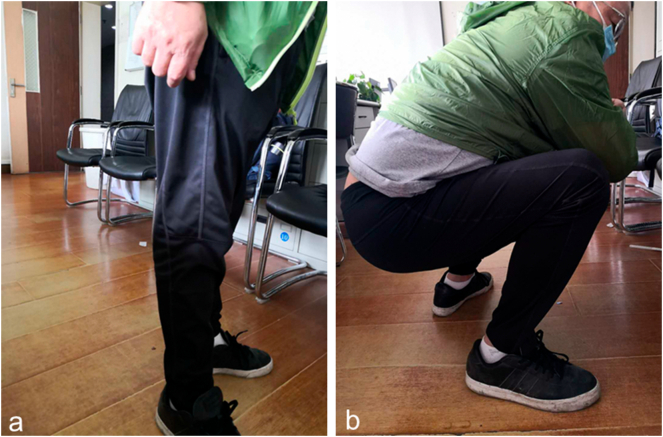
The function of extension and flexion of the injured leg in one year (a, b).

The load-sharing cable was removed in 12 weeks. The physical examination showed the Lachman test (+) and the valgus stress test (−). The X-ray demonstrated slight patella Baja and displacement of the posterolateral tibial plateau fracture was not observed. MRI showed scar healing of the ACL (Fig. 3). At the 12-months follow-up, the range of motion was 0–125° (Fig. 4), and the modified Cincinnati score of the knee achieved 88. No positive finding was noted in Lachman test at the last follow-up.

## Discussion

To our knowledge, the ACL injuries are frequently associated with collateral ligament disruptions. A research based on magnetic resonance imaging demonstrated that, in the setting of an ACL tear in acute knee injuries, the isolated ACL tear occurred at a rate of 61% (83/136) with the second-highest rate being ACL-MCL injuries at 21% (29/136) [3]. In another study, combined ACL-MCL injuries occurred at a rate of 16% within 509 cases with ACL tears [4]. In this injury pattern, the rupture of the ACL often indicates a severe MCL tear. Fetto et al. suggested that among all cases with the MCL tear, approximately 78% of grade III MCL tear consists of mixed ligamentous lesions, with more than 95% of the time involved with the ACL [5]. These injuries are commonly seen, but seldomly associate with the rupture of the PT, which under a typical mechanism of a sudden eccentric contraction of the quadriceps. The traumatic rupture of the PT is rare, only representing 0.6% of all the musculoskeletal tendinous injuries and 5% of all extensor mechanism injuries [6,7]. In sporadic reports published by now, a total of seventeen cases with simultaneous rupture of both the PT and the ACL have been presented, and 5 cases among them were with combined ACL-MCL-PT injuries [2]. The commonest injury pattern was considered to be a sudden anterior tibial translation by a direct impact on the knee followed by an eccentric quadriceps contracture with the knee suddenly flexed and with various degrees of varus or valgus torque. When the knee in flexion, stresses on the extensor mechanism are greater on the patellar tendon than the quadriceps tendon, and the force generated by the quadriceps increase 6% for every degree of the knee flexion [8].

In all types of tibial plateau fractures, the MCL is the most commonly injured ligament with an incidence of 20%, followed by the ACL with an incidence of 10% [9]. In tibial plateau fracture with the posterolateral fragment, as a retrospective review indicates, the incidence of the ACL and the MCL tears was 80% and 60% respectively [10]. Isolated posterolateral tibial plateau fracture with ACL or MCL involved are very rare. Jiang et al. reported two cases with combined ACL rupture and isolated posterolateral tibial plateau fracture and hypothesized the potential injury mechanism follows: the ACL initially ruptured under the force leading to violent internal tibial rotation/anterior tibial translation, followed by the posterolateral tibial plateau fracture stroked by the femoral condyle when the knee was subluxated and internal tibial rotated [11]. Wang et al. reported 3 cases of posterolateral tibial plateau depression fracture associated with rupture of the ACL and MCL, and considered the ACL and MCL injury as a result of the vigorous internal rotation of the tibial or anterior tibial shifting with the knees in a forced valgus mechanism [12]. It is deduced that, in our case, a similar mechanism may play a part, and followed by an eccentric quadriceps' contracture leading to the full tear of the patellar tendon.

There is a possibility of a misdiagnosis of one of these lesions. A high index of suspicion is essential in avoiding missed diagnoses. Routine X-ray examination may not provide sufficient information for the evaluation of the ligamentous damages or non-displaced fracture. The CT and MRI examination is specially indicated when there is no obvious fracture was observed in the X-ray after a severe knee injury. Besides, a meticulous physical examination under anesthesia is compulsory for accurate diagnosis and grading of the damage. Until now, there is no established protocol due to the limited number of reported cases. It is agreed that the PT should be repaired immediately to re-establish the extensor mechanism and start rehabilitation towards restoring range of motion [13]. During the management of the disrupted ACL and MCL, there are controversies around conservative treatment and surgical repair or reconstruction, as well as the timing of surgery [2]. It may be more beneficial to the early activity of patients if a primary repair of the MCL is performed. Under a situation with a full tear of the MCL and PT and moderate rupture of the ACL, it is more recommended to schedule a staged repair in which the MCL and the PT were primarily repaired, followed by the delayed ACL reconstruction, if necessary [14]. Follow the suggestion, we repaired the MCL and PT with augmenting and brace the ACL. However, to avoid further compression of the fracture, the full weight-bearing was not allowed until a month later. The ACL spontaneously healed and the fracture united without further displacement. After 12 months, the patient regains full range of motion and painless walk.

## Conclusion

The involved structures in this rare case consisted of the ACL, the MCL, the PT, and the posterolateral tibial plateau. To this combined injury pattern, a comprehensive consideration, including physical examination, computed tomography, and magnetic resonance imaging, and analysis of injury mechanism, is essential for a full diagnosis and decisioning of the treatment. In treating this compound knee injury, it is suggested to perform a selective or step-by-step repair to the damaged structures, rather than an immediate total repair after injury.

## Declaration of competing interest

The authors declared that they have no conflicts of interest in this work. We declare that we do not have any commercial or associative interest that represents a conflict of interest in connection with the work submitted.

## References

[1] De Baere T., De Muylder J., Deltour A. (2014). An unusual knee trauma: combined rupture of medial collateral ligament and patellar tendon. Case Rep. Orthopedics.

[2] Quinn M., Lemme N., King A. (2019). Simultaneous rupture of the anterior cruciate ligament, medial collateral ligament and patellar tendon: a case series, review of the literature, and proposed treatment algorithm. Int. J. Sports Exercise Med..

[3] LaPrade R.F., Wentorf F.A., Fritts H. (2007). A prospective magnetic resonance imaging study of the incidence of posterolateral and multiple ligament injuries in acute knee injuries presenting with a hemarthrosis. Arthroscopy.

[4] Lee R.J., Margalit A., Nduaguba A. (2018). Risk factors for concomitant collateral ligament injuries in children and adolescents with anterior cruciate ligament tears. Orthop. J. Sports Med..

[5] Fetto J.F., Marshall J.L. (1978). Medial collateral ligament injuries of the knee: a rationale for treatment. Clin. Orthop. Relat. Res..

[6] Clayton R.A., Court-Brown C.M. (2008). The epidemiology of musculoskeletal tendinous and ligamentous injuries. Injury.

[7] Beranger J.S., Kajetanek C., Bayoud W. (2020). Return to sport after early surgical repair of acute patellar tendon ruptures. Orthop. Traumatol. Surg. Res..

[8] Zernicke R.F., Garhammer J., Jobe F.W. (1977). Human patellar-tendon rupture. J. Bone Joint Surg. Am..

[9] Delamarter R.B., Hohl M., Hopp E. (1990). Ligament injuries associated with tibial plateau fractures. Clin. Orthop. Relat. Res..

[10] Wang Y., Cao F., Liu M. (2016). Incidence of soft-tissue injuries in patients with posterolateral Tibial plateau fractures: a retrospective review from 2009 to 2014. J. Knee Surg..

[11] Jiang L., Wu H., Yan S. (2015). Two cases of contact anterior cruciate ligament rupture combined with a posterolateral tibial plateau fracture. Case Rep. Orthop..

[12] Wang Z., Zheng B., Jin Y. (2020). Arthroscopy-assisted surgery: the management of posterolateral tibial plateau depression fracture accompanying ligament injury: a case series and review of the literature. J. Orthop. Surg. (Hong Kong).

[13] Otsubo H., Kamiya T., Suzuki T. (2017). Repair of acute patellar tendon rupture augmented with strong sutures. J. Knee Surg..

[14] Costa-Paz M., Muscolo D.L., Makino A. (2005). Simultaneous acute rupture of the patellar tendon and the anterior cruciate ligament. Arthroscopy.

